# Molecular and Clinical Characteristics of Clonal Complex 59 Methicillin-Resistant *Staphylococcus aureus* Infections in Mainland China

**DOI:** 10.1371/journal.pone.0070602

**Published:** 2013-08-07

**Authors:** Juan Li, Lijuan Wang, Margaret Ip, Mingjiao Sun, Jing Sun, Guoying Huang, Chuanqing Wang, Li Deng, Yuejie Zheng, Zhou Fu, Changcong Li, Yunxiao Shang, Changan Zhao, Sangjie Yu, Kaihu Yao, Yonghong Yang, Xuzhuang Shen

**Affiliations:** 1 Key Laboratory of Major Diseases in Children and National Key Discipline of Pediatrics, Ministry of Education, Beijing Pediatric Research Institute, Beijing Children’s Hospital, Capital Medical University, Beijing, P. R. China; 2 Department of Microbiology, The Prince of Wales Hospital, The Chinese University of Hong Kong, Shatin, Hong Kong; 3 Pediatric Hospital of Fudan University, Shanghai, P. R. China; 4 Guangzhou Children’s Hospital, Guangzhou, P. R. China; 5 Shenzhen Children’s Hospital, Shenzhen, P. R. China; 6 Chongqing Children’s Hospital, Chongqing, P. R. China; 7 Affiliated Hospital of Wenzhou Medical College, Wenzhou, P. R. China; 8 Shenyang Shengjing Hospital, Shenyang, P. R. China; 9 Guangzhou Maternal and Child Health Hospital, Guangzhou, P. R. China; Rockefeller University, United States of America

## Abstract

Detailed molecular analyses of Clonal Complex 59 (CC59) methicillin-resistant *Staphylococcus aureus* (MRSA) isolates from children in seven major cities across Mainland China were examined. A total of 110 CC59 isolates from invasive and non-invasive diseases were analyzed by multilocus sequence typing (MLST), Staphylococcus cassette chromosome mec (SCC*mec*) typing, staphylococcal protein A (*spa*) typing and pulsed-field gel electrophoresis (PFGE). Antibiotics susceptibilities, carriage of plasmids and 42 virulence genes and the expression of virulence factors were examined. ST59 (101/110, 91.8%) was the predominant sequence type (ST), while single locus variants (SLVs) belonging to ST338 (8/110, 7.3%) and ST375 (1/110, 0.9%) were obtained. Three SCC*mec* types were found, namely type III (2.7%), type IV (74.5%) and type V (22.7%). Seven spa types including t437, which accounted for 87.3%, were determined. Thirteen PFGE types were obtained. PFGE types A and B were the major types totally accounting for 81.8%. The dominant clone was ST59-t437-IVa (65.5%), followed by ST59-t437-V (14.5%). The positive rate of *luks-PV and lukF-PV PVL* encoding *(pvl*) gene was 55.5%. Plasmids were detected in 83.6% (92/110) of the strains. The plasmid size ranging from 23.4 kb to 50 kb was most prevalent which accounted for 83.7% (77/92). A significantly lower expression of *hla* was found in ST59-t437-IVa compared with ST59-t437-V. Among the 110 cases, 61.8% of the patients were less than 1 year old. A total of 90 cases (81.8%) were community-associated (CA) infections whereas 20 cases (18.2%) were hospital-associated (HA) infections. Out of the 110 patients, 36.4% (40/110) were diagnosed with invasive infectious diseases in which ST59-t437-IVa accounted for 67.5% (27/40). In brief, ST59-t437-IVa was proved as the dominant clone in CC59 MRSA strains. The carriage rate of *pvl* gene was high. CC59 MRSA could result in CA and HA infections. The majortiy of MRSA infection children were in young age.

## Introduction

Methicillin-resistant *Staphylococcus aureus* (MRSA) infection is an important public health problem worldwide. MRSA causes skin and soft tissues infections, pneumonia and fatal invasive diseases, such as sepsis [1], necrotizing pneumonia [2], necrotizing fasciitis [3], osteomyelitis [4] and orbital cellulitis [5].

Since the first detection of MRSA, different clonal complexes (CCs) have emerged and become the predominant clones in different geographical regions. A majority of hospital-associated (HA)-MRSA strains belong to five clonal complexes, namely CC5, CC8, CC22, CC30 and CC45. In particular, CC5 (ST5), CC8 (ST239) and CC22 (ST22) are distributed globally [6]. CC30 (ST36) is the major clone in the United States and the United Kingdom. Clone type CC45 (ST45) dominates in the United States and the European countries [7]. In Asia, studies have shown that CC5, CC8 and CC22 are the most prevalent clones whilst CC5, CC8 and CC30 are the major clones in Latin America [8]. Community-associated (CA)-MRSA prevalent clones are more diverse compared with HA-MRSA. For instance, CC8 (ST8) is commonly found in the United States and Canada [9]–[11]. CC80 (ST80) is the predominant clone in Europe [12]. ST30 is prevalent in Latin America. In Asia-Pacific areas, CC59 has become the dominant clone while ST5 and ST30 are also prevalent [13] . It is reported that ST59 strains and other CC59 isolates have appeared in other countries, such as Hungary, Denmark, United Kingdom [14], Germany [15] and the United States [16]. A multicenter study of European countries confirmed that the most prevalent MRSA clonal type in Finland, Sweden and Poland is related to the Taiwan clone (ST59-IVa, ST59-V) [17]. CC59 strains from Western Australia have been studied in detail and been differentiated into further subtypes [18]. However, limited studies have emphasized the molecular characteristics of CC59 strains and the clinical traits in large sets of samples. Our previous study showed that the predominant clone in Chinese pediatric community acquired infections was ST59-t437-IVa [8], [19], [20]. The distribution of virulence gene profiles and the presence of exfoliative toxin genes in CA-MRSA have also been determined [21]. In this study, the molecular characteristics of CC59 strains from children in mainland China were described in detail. The expression of virulence factors, the carriage of plasmids and the clinical spectrum of CC59 infections were also elucidated.

## Materials and Methods

### Strains Collection

A total of 299 MRSA isolates were prospectively collected from June 2005 to December 2011. The collection of MRSA covered eight hospitals namely, Beijing Children’s Hospital, Pediatric Hospital of Fudan University, Guangzhou Children’s Hospital, Shenzhen Children’s Hospital, Chongqing Children’s Hospital, Affiliated Hospital of Wenzhou Medical College, Shenyang Shengjing Hospital and Guangzhou Maternal and Child Health Hospital. The first strain isolated from each patient with informed consent was included in the study. The clinical sources of MRSA isolates were sputum, pus, pharyngeal swabs and sterile sites including blood stream, bones and joints, cerebrospinal fluid, lung, pleural cavity, peritoneal cavity and deep seated soft tissue.

Before any study procedures were performed, a parent and/or legal guardian for each participant signed a written informed consent. This study was viewed and approved by the Ethics Committee of Beijing Children’s Hospital Affiliated to Capital Medical University (2008–35).

### Case Definition

MRSA pneumonia was diagnosed based on the medical records, microbiological and radiological results. All the cases exhibited compatible illness with radiological lung infiltrate(s) and MRSA isolated from blood or sputum cultures within two days of hospital admission, or from other sterile respiratory specimen any time during the hospital admission [22]. Severe pneumonia was defined by: (1) a requirement for intensive care unit admission, (2) necrotizing or cavitary infiltrates, or (3) empyema [23].

All the cases with isolates from sterile sites were defined as invasive infections. These included blood, cerebrospinal fluid, pleural fluid, pericardial fluid, peritoneal fluid, joint/synovial fluid, bone and internal body site [24]. Cases meeting the criteria of MRSA severe pneumonia were also included in invasive MRSA infection.

CA-MRSA case was defined as MRSA isolated within 48 h after admission without the following risk factors: history of hospitalization, surgery, dialysis or stay in a long-term care unit within one year; dependence on an indwelling catheter, intravenous line or a percutaneous device when culture; or previous isolation of MRSA, while HA-MRSA case was defined MRSA isolation within 48 h with at least one risk factor or beyond 48 h regardless of risk factors [25].

### DNA Extraction

MRSA was identified as previously described [20]. DNA was extracted from the isolates by using a DNA extraction kit (Saibaisheng, China) and used as the template in all polymerase chain reactions (PCRs) described below.

### Molecular Typing

Multilocus sequence typing (MLST) was performed as described in the previous reports [26]. The allelic profiles were determined based on the database (http://saureus.mlst. net) and the sequence typing (ST) was further analyzed using the eBURST software. STs were subdivided into one group as a clonal complex (CC) only if they were single locus variants (SLV) and shared six identical alleles of seven MLST loci with another ST in the group. The polymorphic X region of the staphylococcal protein A gene (*spa*) was amplified as described previously [27]. The purified *spa* PCR products were sequenced, and compared using the *spa* database website (http://www.ridom.de/spaserver). The staphylococcal cassette chromosome *mec* (SCC*mec*) types were determined using a multiplex PCR assay as described previously [28].The prototypes of SCC*mec* types I to V of the isolates and SCC*mec* IV subtypes were included as controls.

DNA extraction and *SmaI* restriction were performed as described previously [29]. Three *Salmonella* ser. Braenderup H9812 strains digested by *XbaI* were used as the molecular size standard in pulsed-field gel electrophoresis (PFGE) and included in each gel The resulting gel images were analyzed using the guidelines proposed by Tenover [30]. In addition, cluster analysis was performed via the Bio-Numerics software package (Applied Maths, Sint-Martens-Latem, Belgium). Dice coefficient was used and visualized as a dendrogram by using the unweighted pair-group method with the average linkages of 1% tolerance and 1% optimization settings. A similarity cutoff of 80% was used to define a PFGE type while 95% were for a PFGE subtype [20].

### Antimicrobial Susceptibility Testing

The antimicrobial susceptibility profiles were determined and interpreted according to the guidelines of the Clinical and Laboratory Standards Institute (CLSI, 2012). Fifteen antimicrobial agents, including penicilin, erythromycin, clindamycin, cefuroxime, tetracycline, chloramphenicol, gentamicin, ciprofloxacin, sulfamethoxazole-trimethoprim, rifampicin, cefoxitin, vancomycin, linezolid, tigecycline and fusidic acid (Sigma) were used. The breakpoints for Fusidic acid and tigecycline were based on the European Committee on Antimicrobial Susceptibility Testing (EUCAST, Version 3.1, 2013) guidelines. Mupirocin was obtained from the National Institute for the Control of Pharmaceutical and Biological Products. The isolates with an MIC from 8 µg/ml to 256 µg/ml were regarded as low-level mupirocin resistant whereas those with the MIC of ≥512µg/ml were considered as high-level mupirocin resistant. *Staphylococcus aureus* ATCC 29213 was used as control for each set of tests.

### Plasmids Detection

PFGE was performed using total DNA of the strains without lysis by *SmaI* to detect plasmids [31]. A lambda DNA (Saibaisheng, China) molecular size standard was included in each gel.

### Screening of 42 Virulence Genes

The isolates were screened for 42 known virulence genes by PCRs using conserved primers as previously described and listed in Table S1 in file S1 [32]–[39]. The presence of the 21 known SAg genes (enterotoxin genes: *sea*, *seh*, *sec*, *sed*, *sek*, *see*, *seb*, *sem*, *sel*, *seo*, *sen*, *seg*, *seq*, *sej*, *sei*, *seu*, *ser* and *sep*; toxic shock syndrome toxin gene *tsst-1*, two exfoliative toxin genes: *eta* and *etd*), and the *agr* types were tested by six multiplex PCRs. The multiplex PCRs included multiplexes I (*sea*, *seh*, *sec*, and *tsst-1*), II (*sed*, *etd*, *eta* and *sek*), III (*see*, *seb*, *sem*, *sel* and *seo*), IV (*sen*, *seg*, *seq*, and *sej*), V (*sei*, *ser*, *seu* and *sep*) and VI (*agr*-1 to *agr*-4). The remaining genes, including exfoliative toxin B gene (*etb*), fibronectin-binding protein genes (*fnbA* and *fnbB*), clumping factor genes (*clfA* and *clfB*), bone sialoprotein gene (*bbp*), collagen-binding protein gene (*cna*), extracellular adherence protein gene(*eap*), elastin-binding protein gene (*ebpS*), genes for the serine-aspartate repeat family of proteins (*sdrC*, *sdrD* and *sdrE*), *spa* gene, hemolysin genes (*hla*, *hlb*, *hld*, *hlg* and *psmα*) and leukocidin genes (*luks-PV and lukF-PV PVL* encoding gene, *lukD* and *lukE*) were screened by singleplex PCR.

A positive control and a negative control were included for each respective gene in reach run. A positive PCR was determined by the presence of an appropriately sized DNA band with the positive control strain in the gel. Two PCR products per gene were randomly chosen for DNA sequencing for validation.

### RNA Isolation and Complementary DNA (cDNA) Synthesis

The overnight cultures were diluted 1∶100 in 10 mL of tryptic soy broth (TSB) and incubated at 37°C with shaking at 220 rpm until stationary growth phase was reached (appropriately 6 h). The cell aliquots harvested during the stationary phase were pelleted by centrifugation at 12,000 rpm at −4°C for 5 min. Each pellet was washed in an equal volume of Tris-EDTA (TE) buffer [10 mM TrisHCl, 1 mM EDTA (pH 8)] and resuspended in TE buffer (pH 8.0) containing 10 g/L lysozyme and 40 mg/L lysostaphin and then incubated at 37°C for 15 min. The total bacterial RNA was isolated using RNAiso (Takara, Japan) according to the manufacturer’s instructions. The contaminating DNA was removed by incubating the total bacterial RNA with RNase-free DNase I (30 U/100 µg of total RNA, Takara, Japan) at 37°C for 1 h. The amount of recovered RNA was determined using a NanoDrop fluorospectrometer (NanoDrop Technologies, Wilmington, DE, USA). The absence of DNA was verified by PCR. The samples were then stored at −80°C. Complementary DNA (cDNA) was synthesized from the total RNA by using the PrimeScript RT reagent kit (Takara, Japan) according to the manufacturer’s instructions. The recovered cDNA was quantitated spectrophotometrically.

### Quantitative Real-time (qRT-) PCR

qRT-PCRs were performed using SYBR Premix Ex Taq™ (Takara, Japan) according to the instructions. Real-time detection and relative quantitation were performed using the Bio-rad CFX96 PCR Detection System. The selected genes were analyzed using the primers shown in Table 1. As an endogenous control, primers were used to amplify a 91 bp fragment of the DNA gyrase (gyrB) [33]. The calibrators in this study were the transcripts from the MRSA strains N315 and GZ11 [40]. Relative quantification was conducted using the ΔΔCT method, with the expression of N315 as the reference of *hla*, *psmα*, and *RNAIII*. GZ11 was used as the reference of *pvl*. Relative quantification was expressed as the n-fold difference relative to the reference. The qRT-PCR assays were performed in triplicate.

**Table 1:** PFGE, MLST, *spa*, SCC*mec* types and *pvl* results of CC59 MRSA isolates.

PFGE pattern	No.(%) of isolates	MLST allele No.	MLST	*spa* allele No.	*spa*	SCC*mec*	No. of isolates	*pvl* (+) No.
A	45(40.9)	19-23-15-2-19-20-15	59	04-20-17-20-17-25-34	t437	IVa	32	6
		19-23-15-2-19-20-15	59	04-20-17-20-17-25-34	t437	V	5	1
		19-23-15-2-19-20-15	59	04-20-17-25-34	t441	IVa	1	0
		19-23-15-2-19-20-15	59	04-20-17-20-17-25-34-34	t3523	IVa	2	2
		19-23-15-2-19-20-15	59	04-20-17-20-17-20-17-25-34	t3485	IVa	2	1
		19-23-15-2-19-20-15	59	NT	NT	III	1	0
		19-23-15-2-19-20-15	59	04-20-17-20-17-25-34	t437	III	1	0
		19-23-15-2-19-20-42	375	15-12-16-02-24-24-24	t2270	IVa	1	0
B	45(40.9)	19-23-15-2-19-20-15	59	04-20-17-20-17-25-34	t437	IVa	27	25
		19-23-15-2-19-20-15	59	04-20-17-20-17-25-34	t437	V	9	8
		19-23-15-2-19-20-15	59	04-20-17-25-34	t441	IVa	1	1
		19-23-15-2-19-20-15	59	121-21-51-17-17-17-23-24	t5350	V	1	1
		19-23-15-48-19-20-15	338	04-20-17-20-17-25-34	t437	V	4	3
		19-23-15-48-19-20-15	338	04-20-17-20-17-25-34	t437	III	1	1
		19-23-15-48-19-20-15	338	04-02-17-20-17-25-34	t3590	V	2	2
C	6(5.5)	19-23-15-2-19-20-15	59	04-20-17-20-17-25-34	t437	IVa	4	0
		19-23-15-2-19-20-15	59	04-20-17-20-17-25-34	t437	IVc	1	0
		19-23-15-2-19-20-15	59	04-20-17-25-34	t441	IVa	1	0
D	4(3.6)	19-23-15-2-19-20-15	59	04-20-17-20-17-25-34	t437	IVa	3	3
		19-23-15-2-19-20-15	59	04-20-17-25-34	t441	IVa	1	1
E	2((1.8)	19-23-15-2-19-20-15	59	04-20-17-20-17-25-34	t437	IVa	1	0
		19-23-15-2-19-20-15	59	04-20-17-20-17-25-34	t437	V	1	0
F	1(0.9)	19-23-15-2-19-20-15	59	04-20-17-20-17-25-34	t437	IVa	1	1
G	1(0.9)	19-23-15-2-19-20-15	59	04-20-17-25-34	t441	IVa	1	1
H	1(0.9)	19-23-15-2-19-20-15	59	04-20-17-20-17-25-34-34	t3523	IVa	1	0
I	1(0.9)	19-23-15-2-19-20-15	59	04-20-17-20-17-25-34	t437	IVa	1	1
J	1(0.9)	19-23-15-2-19-20-15	59	04-20-17-20-17-25-34	t437	IVa	1	1
K	1(0.9)	19-23-15-2-19-20-15	59	04-20-17-20-17-25-34	t437	V	1	1
L	1(0.9)	19-23-15-2-19-20-15	59	04-20-17-20-17-25-34	t437	V	1	0
M	1(0.9)	19-23-15-48-19-20-15	338	04-20-17-20-17-25-34	t437	V	1	1

### Statistical Analysis

Statistical analysis was performed using the SPSS 16.0 software. The categorical variables were compared using the chi-squared or two-tailed Fisher’s exact test. The mean of two independent samples were compared using Student’s *t*-test. *P* values <0.05 were considered statistically significant.

## Results

### Molecular Characteristics of CC59 Strains

**Figure 1:**
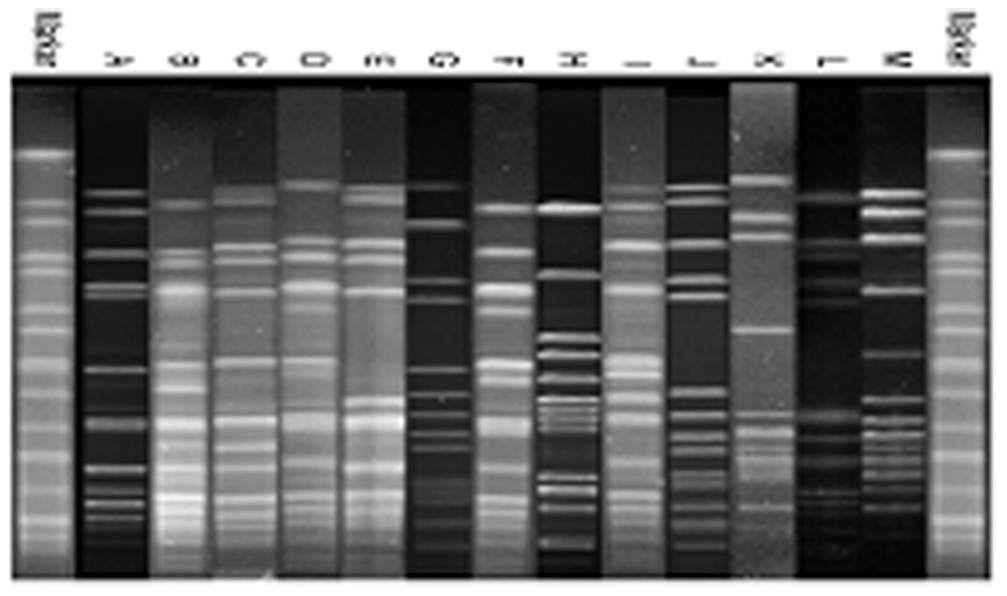
Thirteen representative pulsed-field gel electrophoresis patterns of the 110 CC59 MRSA strains. Lanes marker, chromosomal DNA of *Salmonella* ser. Braenderup strain H9812 (27) was used as the size marker. (A) SZ12 MRSA ST59-t437-IVa; (B) G20 MRSA ST59-t437-IVa; (C) CQ6 MRSA ST59-t437-IVa; (D) BJ313 MRSA ST59-t437-IVa; (E) SH35 MRSA ST59-t437-V; (F) BJ57 MRSA ST59-t437-IVa; (G) SY183 MRSA ST59-t441-IVa; (H) BJ326 MRSA ST59-t3523-IVa; (I) SZ46 MRSA ST59-t437-IVa; (J) BJ787 MRSA ST59-t437-IVa; (K) SY76 MRSA ST59-t437-V; (L) BJ860 MRSA ST59-t437-V and (M). GZ1 MRSA ST338-t437-V.

According to the MLST analysis, 110 isolates were defined as CC59 including 101 ST59 strains (91.8%), 8 ST338 strains (7.3%) and 1 ST375 strain; the latter two being SLVs of ST59. Thirteen PFGE types without subtypes were obtained (Figure 1). PFGE types A and B were the major types, representing 40.9% (45/110) each of types A and B respectively. These strains belonged to one of three SCC*mec* types (types III, IV and V); with SCC*mec* IV identified as the dominant SCC*mec* type (74.5%, 82/110 strains, 81 were SCC*mec* IVa and 1 was SCC*mec* IVc). SCC*mec*V and SCC*mec* III were present in 25 strains (22.7%) and 3 strains (2.7%) respectively. Seven spa types were obtained and the dominant type was t437 accounting for 87.3% of all strains. The other types were t441, t3523, t3590, t3485, t5350 and t2270 which accounted 4.5%, 2.7%, 1.8%, 0.9%, 0.9% and 0.9% respectively. One NT strain was also found. The dominating clone was ST59-t437-IVa (65.5%), followed by ST59-t437-V (14.5%) on the basis of MLST, spa and SCC*mec* types. Furthermore, ST59-t441-IVa (4.5%) and ST338-t437-V (4.5%) were obtained (Table 1). The total carriage rate of the *pvl* gene was 55.5% (61/110).

A significantly higher carriage of *pvl* gene was found in PFGE type B in comparison with PFGE type A strains. The carriage of *pvl* gene accounted for 91.1% (41/45) and 22.2% (10/45) respectively (*P*<0.01) of these PFGE types. The prevalence of *pvl* gene in ST59-t437-IVa and ST59-t437-V were 51.4% (37/72) and 62.5% (10/16) respectively.

### Antibiotic Resistance of CC59 Strains

All of the strains were susceptible to vancomycin, linezolid, tigecycline and fusidic acid. Various resistant rates were obtained for the other antibiotics. The percentage of isolates resistant to various antibiotics is summarized in Table 2. Two strains exhibited low-level resistance to mupirocin. A significant difference was found in the rate of resistance to chloramphenicol between PFGE types A and B (20% vs. 41%, *P*<0.05). The rate of resistance to chloramphenicol of ST59-t437-IVa was obviously lower than that of ST59-t437-V (26.4% vs. 56.3%, *P*<0.05). In contrast, the percentage of isolates resistant to ciprofloxacin was higher than that in ST59-t437-V (38.9% vs. 12.5%, *P*<0.05).

**Table 2:** Resistance to 16 antimicrobial agents of CC59 isolates in the major PFGE groups and predominant clones.

Antimicrobialagents	CC59(n = 110)			PFGE A group(n = 45)			PFGE B group(n = 45)			P* value	ST59-t437-IVa(n = 72)			ST59-t437-V(n = 16)			P** value
	% aS	% bR	% cI	% S	% R	% I	% S	% R	% I		% S	% R	% I	% S	% R	%I	
Cefoxitin	0.0	100.0	0.0	0.0	100.0	0.0	0.0	100.0	0.0		0.0	100.0	0.0	0.0	100.0	0.0	
Penicillin	2.9	97.1	0.0	2.2	97.8	0.0	2.3	97.7	0.0		1.4	98.6	0.0	6.2	93.8	0.0	
Cefuroxime	33.0	67.0	0.0	37.8	62.2	0.0	30.0	70.0	0.0		15.3	84.7	0.0	25.0	75.0	0.0	
Gentamicin	78.6	21.4	0.0	84.4	15.6	0.0	81.8	18.2	0.0		79.2	20.8	0.0	62.5	37.5	0.0	
Chloramphenicol	67.0	33.0	0.0	80.0	20.0	0.0	59.0	41.0	0.0	<0.05	73.6	26.4	0.0	43.7	56.3	0.0	<0.05
Tetracycline	36.0	44.6	19.4	44.4	35.6	20.0	29.5	52.3	18.2		10.6	83.3	6.1	6.2	93.8	0.0	
Sulfamethoxazole-trimethoprim	89.3	10.7	0.0	95.6	4.4	0.0	86.4	13.6	0.0		84.7	15.3	0.0	87.5	12.5	0.0	
Rifampicin	89.3	10.7	0.0	93.3	6.7	0.0	84.1	15.9	0.0		91.7	8.3	0.0	81.2	18.8	0.0	
Erythromycin	0.9	99.1	0.0	0.0	100.0	0.0	0.0	100.0	0.0		0.0	100.0	0.0	0.0	100.0	0.0	
Clindamycin	6.4	93.6	0.0	8.9	91.1	0.0	2.3	97.7	0.0		4.2	95.8	0.0	6.2	93.8	0.0	
Ciprofloxacin	75.7	11.7	12.6	68.9	8.9	22.2	81.8	11.4	6.8		47.2	38.9	13.9	87.5	12.5	0.0	<0.05
Vancomycin	100.0	0.0	0.0	100.0	0.0	0.0	100.0	0.0	0.0		100.0	0.0	0.0	100.0	0.0	0.0	
Linezolid	100.0	0.0	0.0	100.0	0.0	0.0	100.0	0.0	0.0		100.0	0.0	0.0	100.0	0.0	0.0	
Tigecycline	100.0	0.0	0.0	100.0	0.0	0.0	100.0	0.0	0.0		100.0	0.0	0.0	100.0	0.0	0.0	
Fusidic acid	100.0	0.0	0.0	100.0	0.0	0.0	100.0	0.0	0.0		100.0	0.0	0.0	100.0	0.0	0.0	
Mupirocin	98.2	1.8	0.0	100.0	0.0	0.0	100.0	0.0	0.0		98.6	1.4	0.0	100.0	0.0	0.0	

a S = susceptible.

b R = resistant.

c I = intermediate.

P* value: The resistant rate between PFGE A and B groups was compared.

P** value: The resistant rate between ST59-t437-IVa and ST59-t437-V was compared.

### Plasmid Patterns of CC59 Strains

Plasmids were detected in 83.6% (92/110) of the strains (Figure S1). Eighty five strains (85/92, 92.4%) carried a single plasmid while seven strains (7/92, 7.6%) contained more than one plasmid. In the single plasmid profile, plasmid ranging in size of 23.4 Kb to 50 Kb accounted for 90.6% (77/85), followed by the plasmid of <4 kb and 4 kb to 23.4 kb which accounted for 4.7% (4/85) each. The prevalence of the plasmid in PFGE types A and B as well as ST59-t437-IVa and ST59-t437-V were 84.4% (38/45), 82.2% (37/45) and 86.1% (62/72) and 93.8% (15/16) respectively.

### Toxin Genes Content and Expression of hla, psmα, pvl, and RNAIII

All of the 110 strains harbored these nine virulence genes, namely *fnbA*, *clfA*, *clfB*, *ebpS*, *sdrC*, *spa*, *psmα*, *hla* and *hlb*. Fifteen toxin genes, including *sed*, *sem*, *sel*, *seo*, *sen*, *seg*, *sej*, *sei*, *seu*, *ser*, *sep*, *eta*, *etb*, *etd* and *lukM*, were not detected. The percentage of positivity of individual superantigen (SAg) genes was variable in the order as follows: *seb* = *seq*>*sek*>*sea*>*sec* = *tst*>*she*>*see* (Figure S2). For adhesion genes, the carriage rates of *bbp*, *sdrE*, *sdrD*, *eap*, *fnbB* and *can* were 97.5%, 97.5%, 0.9%, 63.6%, 5.6% and 0.9% respectively. For hemolysin genes, *hld* and *hlg* were found in 98.4% of the strains. For leukocidin genes, 33.3% of the strains carried *lukE* gene. Agr types were analyzed and all of the strains were identified as agr type I. Of all strains, 93.6% (103/110) contained at least one SAg gene. *seb-sek-seq* was the most common gene profile which was harbored by 46 strains (44.7%, 46/103). *sea*-*seb*-*sek*-*seq* was also detected in 30 strains (29.1%, 30/103) (Figure S2). A significant difference was observed in the carriage of *lukE* gene in PFGE types A and B (15% vs. 50%, *P*<0.05). Six strains in PFGE type B carried *fnbA* and *fnbB* genes simultaneously, in which 5 belonged to ST59-t437-IVa and 1 belonged to ST338-t437-III. In contrast to ST59-t437-V, *see* and *fnbB* genes were only detected in 5% and 8.3% of clone ST59-t437-IVa. No significant difference was found between ST59-t437-IVa and ST59-t437-V in term of the remaining of toxin genes.

The expressions of *hla*, *psmα*, *pvl* and *RNAIII* are shown in Figure S3. No significant difference was observed between PFGE types A and B in the expression of *hla*, *psmα*, *pvl* and *RNAIII*. A significantly lower expression of *hla* was found in ST59-t437-IVa compared with ST59-t437-V. No significant difference was found in the expressions of *psmα*, *pvl* and *RNAIII* in these two major clones.

### Clinical Features

Of 110 cases, 90 cases were epidemiologically defined as CA-MRSA cases and 20 classified as HA-MRSA cases. Sixty-seven patients (60.9%) were male. The median age was 4.8 months. 61.8% (68/110) of the children were <1-year old and 23 were neonates (20.9%). The distribution of MRSA ST-spa-SCCmec types and the corresponding clinical spectrum of diseases among CA- and HA-MRSA cases are listed in Table S2 in file S1. ST59-t437-IVa was the major clone and accounted for 66.7% (60/90) and 60% (12/20) of CA- and HA-MRSA cases respectively. Forty (36.4%) cases presented with invasive diseases including severe pneumonia, bacteremia, meningitis, osteomyelitis, cellulitis with bacteremia, suppurative peritonitis and arthritis. The clone type ST59-t437-IVa was also the predominant cause of invasive disease and contributed to 67.5% (27/40) of these cases whereas ST59-t437-V contributed to only 7.5% (3/40). For non-invasive diseases, ST59-t437-IVa and ST59-t437-V accounted for 64.3% (45/70) and 18.6% (13/70) respectively. For CA infections and HA infections, invasive infectious disease accounted for 32.2% and 55% respectively. Eight cases with ST338 strains were identified: five cases with skin and soft tissue infections, one case with suppurative peritonitis, one case with severe pneumonia and one case with colonization. ST375 was found in one case with pneumonia which was listed as a non-invasive infectious disease.

## Discussion

This study was the first to report the detailed molecular characteristics of CC59 MRSA strains from children in mainland China and described the clinical spectrum of disease in relation to the strain types identified. Since the first report of MRSA in 1959 [41], more than 20 sporadic clones associated with the distribution of CA-MRSA have emerged, including ST59-V (Taiwan clone). In the description of CC59 strains from Western Australia, ST59-t437-V and ST59-t976-IVa are reported as the predominant clones [18] whilst in Taiwan, ST59-t437-V was the most common [42]–[45]. In this study, ST59-t437-IVa was found as the major clone in CC59 strains of children from mainland China, suggesting geographical differences existed in adjacent regions. Based on the results of spa typing, seven types were received in CC59 strains and the distribution was concentrated in spa t437 and t441 which accounted for over 90% of all the strains. Several spa types shared the spa alleles according to the sequence of spa alleles compared with the dominant type t437 such as t441, t3590, t3523 and t3485. Although the strains belonged to the same CC clone, the genetic background remained variable. In this study 13 diverse PFGE types were obtained from 110 CC59 strains.

The prevalence of *pvl* gene among MRSA in different regions is diverse. In Belgium 39% of the strains are *pvl* positive based on a study of 410 CA-MRSA strains [46]. In Europe, the positive strains accounted for only 1.1% in a research of MRSA isolated from invasive infections [47]. In Asia, the prevalence of *pvl* gene in CA and HA-MRSA are 14.3% and 5.7% respectively [13]. In Taiwan, the data was also different. In a research of 253 MRSA strains from bloodstream infection [48], the percentage of *pvl* positive strains was 11.1%. In a study of 126 community-onset MRSA bacteremia, 7.1% of the strains are detected *pvl* gene. In a study of 302 MRSA isolates from adults in mainland China, ST239 is the predominant clone and only 2 strains are found with *pvl* gene, suggesting that various clones may influence the carriage rate of *pvl* gene. In this study, the carriage rate of *pvl* gene in CC59 strains was 55.5%, which was higher than the carriage rates presented in the aforementioned reports.

Plasmids are the vital mobile genetic elements (MGEs) in the carriage and dissemination of antibiotic resistance genes. The studies of plasmids carriage provide evidence that illustrates the transmission of plasmids between different clones or within one clone and reveals the possible routes of plasmid dissemination. The carriage rates of plasmid in MRSA determined in previous reports differ ranging from 61.2% to 100% [49], [50]. The carriage rate of plasmids in our study was high up to 83.6%. In a study involving 73 clinical strains from Czech, 44% of the strains contain a single plasmid whereas 45% of the isolates contain more than one plasmid [51]. In our study, 92.4% of the strains carried a single plasmid. Plasmid of 23.4 kb to 50 kb was the most common which accounted for 90.6%. Previously we showed that the position of *mup*A gene in high level mupirocin resistant MRSA was present in the plasmid of 23.4 kb to 50 kb [52]. Further studies are needed to explain the relationship between antibiotic resistant genes and plasmids. Currently, all of the CC59 strains were susceptible to vancomycin, linezolid, tigecycline and fusidic acid.

This study was the first to describe the expression of relevant virulence factors in CC59 strains. Although the clone ST59-t437-IVa was the predominant clone to cause severe disease, the expression of *hla* in ST59-t437-IVa was distinctly lower than that in ST59-t437-V whilst no difference was found in the expression of *psmα*, *pvl* and *RNAIII*. It is likely that the expressions of *hla*, *psmα*, *pvl* and *RNAIII* are intricately regulated and their roles in virulence remain poorly elucidated.

The carriage of SAg genes was non-uniform even if the strains were isolated from the same CC. In a description of CC59 MRSA strains obtained from New York, *ser* gene is the dominant SAg gene [53]. In the current study, *sek* and *seq* genes were the prevalent SAg genes in CC59. Certain SE genes are usually grouped together on MGEs. The genes of *sea-sek-seq* are typically located in phageφ3 [54]. A family of *seb*-*sek*-*seq* is present on the pathogenicity islands (SaPI) [55]. A group of enterotoxin gene cluster (egc cluster, *seg*-*sei*-*sem*-*sen*-*seo*) is found on the genomic island vSAb [55]. The results in our research implied the co-existence of SaPI and phageφ3 without the genomic island vSAb, which suggested some MGEs might influence the acquisition of other MGEs. In addition, Brazil clone BEC/ST239 is the first lineage carrying both *fnb*A and *fnb*B genes [56]. In this study, six strains carrying both *fnb*A and *fnb*B genes were found. Among these strains, five strains belonged to ST59-t437-IVa and one belonged to ST338-t437-III. However, studies on the explanation of adhesion genes in CC59 strains are limited. The carriage of *fnb*A and *fnb*B genes might benefit the transmission of CC59 strains to some extent. To the best of our knowledge, this study was the first to report the carriage of *fnb*A and *fnb*B in CC59 strains.

Few reports have discribed the clinical traits of CC59 infection cases in the pediatric population. The majority of pediatric MRSA infections (61.8% ) described in this study were found in neonates and infants. Invasive disease was prevalent due to the immature immune system in this age, and possiblely the pathogenicity of CC59 strains. Besides, ST59 is previously prevalent in the community of China [19], [20], [57]. In this study ST59-t437-IVa accounted for 67% of CA infections and the same clone accounted for 60% in HA infections. Our resluts revealed that 18.2% of CC59 strains were HA-MRSA suggesting that the distinction of strains types between CA- and HA-MRSA are becoming unclear. In Taiwan, reports of local community associated clone ST59 has been introduced into hospital, causing hospital associated infections [58], [59]. Numbers of reports even suppose that CA-MRSA would replace HA-MRSA gradually in future becaues of the small SCC*mec*(usually SCC*mec* types IV and SCC*mec*V) in CA-MRSA, resulting in increased adaptability and shortened doubling time compared with HA-MRSA [60]. Further monitoring of these CA-MRSA genotypes would be necessary to determine if they would replace the traditional HA-MRSA types in Chinese children.

This study provided the baseline data of the molecular traits and clinical characteristics of CC59 MRSA infections in children in mainland China. The result showed that ST59-t437-IVa was the dominant clone and infections among the pediatric population were mainly among young children. The carriage rate of *pvl* gene was high. Most strains carried one single plasmid ranging from 23.4 kb to 50 kb. Strains type of CC59 MRSA, the local CA-MRSA, is also found associated with hospital cases.

## Supporting Information

Figure S1 **Distribution of plasmids among CC59 isolates.** Lane marker, λDNA/Hind III (Tiangen) was used as size marker. Lane 5, carries 4 to 23 kb plasmid; Lane 8, carries 4–23 kb plasmid and <4 kb plasmid; Lanes 9,13 and 14 carry <4 kb plasmid; Lanes 1, 2, 3, 4, 6, 7, 10, 11 and 12 carry no plasmid.
(TIF)

Figure S2 **Distribution of virulence genes in 110 CC59 isolates.** A: Percentage of SAg genes no carried in per isolates. B: Percentage of 27 toxin genes carried in total CC59 isolates. *sed, sem, sel, seo, sen, seg, sej, sei, seu, ser, sep, eta, etb, etd* and *lukM* genes were not listed in the figure for no carriage in all isolates.
(TIF)

Figure S3 **Expression levels of hla, psmá, pvl and RNAIII in CC59 strains.** Expression levels of *hla, psmα, pvl* and *RNAIII* in different PFGE patterns and predominant clones were measured by qRT-PCR until reached to the stationary growth phase in TSB. N315 and GZ11 were used as the normalized control to measure the sample expression. The results denote the means of every group and are presented as means± standard errors. *P*<0.05 represents the statistical value of the expression of hla.
(TIF)

File S1 Table S1, Primers used in this study. Table S2, Clinical characteristics and molecular typing of CC59 isolates.
(DOCX)
