# Supplementary figures and images for: Molecular and Clinical Characteristics of Clonal Complex 59 Methicillin-Resistant Staphylococcus aureus Infections in Mainland China
Source: PLoS One. 2013 Aug 7;8(8):e70602. doi: 10.1371/journal.pone.0070602 (PMC3737374; doi:10.1371/journal.pone.0070602)

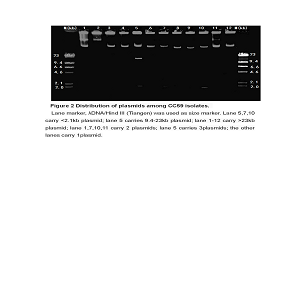

Supplement: Figure S1 — Distribution of plasmids among CC59 isolates. Lane marker, λDNA/Hind III (Tiangen) was used as size marker. Lane 5, carries 4 to 23 kb plasmid; Lane 8, carries 4–23 kb plasmid and <4 kb plasmid; Lanes 9,13 and 14 carry <4 kb plasmid; Lanes 1, 2, 3, 4, 6, 7, 10, 11 and 12 carry no plasmid. (TIF) [file pone.0070602.s001.tif]

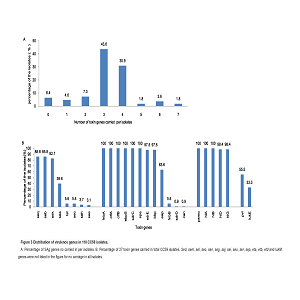

Supplement: Figure S2 — Distribution of virulence genes in 110 CC59 isolates. A: Percentage of SAg genes no carried in per isolates. B: Percentage of 27 toxin genes carried in total CC59 isolates. sed, sem, sel, seo, sen, seg, sej, sei, seu, ser, sep, eta, etb, etd and lukM genes were not listed in the figure for no carriage in all isolates. (TIF) [file pone.0070602.s002.tif]

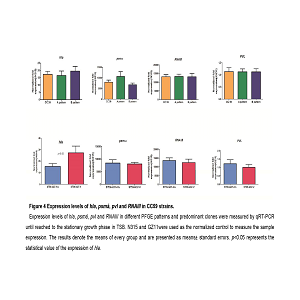

Supplement: Figure S3 — Expression levels of hla, psmá, pvl and RNAIII in CC59 strains. Expression levels of hla, psmα, pvl and RNAIII in different PFGE patterns and predominant clones were measured by qRT-PCR until reached to the stationary growth phase in TSB. N315 and GZ11 were used as the normalized control to measure the sample expression. The results denote the means of every group and are presented as means± standard errors. P<0.05 represents the statistical value of the expression of hla. (TIF) [file pone.0070602.s003.tif]
