# Supplementary material for: Molecular and Clinical Characteristics of Clonal Complex 59 Methicillin-Resistant Staphylococcus aureus Infections in Mainland China
Source: PLoS One. 2013 Aug 7;8(8):e70602. doi: 10.1371/journal.pone.0070602 (PMC3737374; doi:10.1371/journal.pone.0070602)
Supplement: File S1 — Table S1, Primers used in this study. Table S2, Clinical characteristics and molecular typing of CC59 isolates. (DOCX) [file pone.0070602.s004.docx]

**Table S1. Primers used in this study**

| Gene | | Forward primer  (5'-3') | Reverse primer (5'-3') | | | Fragment size (bp) | Control strain/ GenBank no. | |
| --- | --- | --- | --- | --- | --- | --- | --- | --- |
| Multiplex I | *sea* | GAAAAAAGTCTGAATTGCAGGGAACA | CAAATAAATCGTAATTAACCGAAGGTTC | | | 560 | FRI913 | |
|  | *seh* | CAATCACATCATATGCGAAAGCAG | CATCTACCCAAACATTAGCACC | | | 376 | FRI137 | |
|  | *sec* | CTTGTATGTATGGAGGAATAACAAAACATG | CATATCATACCAAAAAGTATTGCCGT | | | 275 | FRI913 | |
|  | *tsst-1* | TTCACTATTTGTAAAAGTGTCAGACCCACT | TACTAATGAATTTTTTTATCGTAAGCCCTT | | | 180 | FRI913 | |
| MultiplexII | *sed* | GAATTAAGTAGTACCGCGCTAAATAATATG | GCTGTATTTTTCCTCCGAGAGT | | | 492 | FRI1151m | |
|  | *etd* | CAAACTATCATGTATCAAGGATGG | CCAGAATTTCCCGACTCAG | | | 358 | TY114 | |
|  | *eta* | ACTGTAGGAGCTAGTGCATTTGT | TGGATACTTTTGTCTATCTTTTTCATCAAC | | | 190 | A920210 | |
|  | *sek* | ATGCCAGCGCTCAAGGC | AGATTCATTTGAAAATTGTAGTTGATTAGCT | | | 134 | FRI913 | |
|  |  |  | TGCCAGCGCTCAAGGTG | | |  |  | |
| MultiplexIII | *see* | CAAAGAAATGCTTTAAGCAATCTTAGGC | CACCTTACCGCCAAAGCTG | | | 482 | FRI918 | |
|  | *seb* | ATTCTATTAAGGACACTAAGTTAGGGA | ATCCCGTTTCATAAGGCGAGT | | | 404 | CCM5757 | |
|  | *sem* | CTATTAATCTTTGGGTTAATGGAGAAC | TTCAGTTTCGACAGTTTTGTTGTCAT | | | 326 | FRI137 | |
|  | *sel* | GCGATGTAGGTCCAGGAAAC | CATATATAGTACGAGAGTTAGAACCATA | | | 234 | FRI137 | |
|  | *seo* | AGTTTGTGTAAGAAGTCAAGTGTAGA | ATCTTTAAATTCAGCAGATATTCCATCTAAC | | | 180 | FRI137 | |
| MultiplexIV | *sen* | CGTGGCAATTAGACGAGTC | GATTGATYTTGATGATTATKAG | | | 474 | FRI137 | |
|  | *seg* | TCTCCACCTGTTGAAGG | AAGTGATTGTCTATTGTCG | | | 323 | FRI137 | |
|  | *seq* | ACCTGAAAAGCTTCAAGGA | CGCCAACGTAATTCCAC | | | 204 | COL | |
|  | *sej* | TCAGAACTGTTGTTCCGCTAG | GAATTTTACCAYCAAAGGTAC | | | 138 | FRI1151m | |
| MultiplexV | *sei* | CTYGAATTTTCAACMGGTAC | AGGCAGTCCATCTCCTG | | | 461 | FRI137 | |
|  | *ser* | AGCGGTAATAGCAGAAAATG | TCTTGTACCGTAACCGTTTT | | | 363 | FRI1151m | |
|  | *seu* | AATGGCTCTAAAATTGATGG | ATTTGATTTCCATCATGCTC | | | 215 | FRI137 | |
|  | *sep* | GAATTGCAGGGAACTGCT | GGCGGTGTCTTTTGAAC | | | 182 | N315 | |
| MultiplexVI | *agr1–4*  *pan agr* ATGCACATGGTGCACATGC | |  | | |  |  | |
|  | *agr-1* |  | GTCACAAGTACTATAAGCTGCGAT | | | 439 | COL | |
|  | *agr-2* |  | TATTACTAATTGAAAAGTGCCATAGC | | | 572 | N315 | |
|  | *agr-3* |  | GTAATGTAATAGCTTGTATAATAATACCCAG | | | 320 | TY114 | |
|  | *agr-4* |  | CGATAATGCCGTAATACCCG | | | 657 | A920210 | |
|  | *etb* | CAGATAAAGAGCTTTATACACACATTAC | AGTGAACTTATCTTTCTATTGAAAAACACTC | | | 612 | TY4 | |
|  | *clfA* | TGCTGCACCTAAAACAGACG | CCTCCGCATTTGTATTGCTT | | | 230 | MW2 | |
|  | *clfB* | GCTGCAAAAATGCAAGATCA | CGCATTGGAAATTGTTTGTG | | | 159 | MW2 | |
|  | *fnbA* | GCGGAGATCAAAGACAA | CCATCTATAGCTGTGTGG | | | 1279 | MW2 | |
|  | *fnbB* | GGAGAAGGAATTAAGGCG | GCCGTCGCCTTGAGCGT | | | 812 | COL | |
|  | *bbp* | TCAAAAGAAAAGCCAATGGCAAACG | ACCGTTGGCGTGTAACCTGCTG | | | 500 | NRS71 | |
|  | *cna* | AGCATTTGCAGCACGAGATA | CTTGACCCACCTGTTCACCT | | | 233 | ATCC25923 | |
|  | *eap* | GCATGATAGAGGTATCGGGGAACGTG | TCCCTTGATCATTTGCCATTGCTG | | | 655 | ATCC25904Newman | |
|  | *ebpS* | GCAAGTAATAGTGCTTCTGCCGCTTCA | CATTTTCCGGTGAACCTGAACCGTAGT | | | 550 | NRS71 | |
|  | *sdrC* | CGCATGGCAGTGAATACTGTTGCAGC | GAAGTATCAGGGGTGAAACTATCCACAAATTG | | | 731 | ATCC25904Newman | |
|  | *sdrD* | CCACTGGAAATAAAGTTGAAGTTTCAACTGCC | CCTGATTTAACTTTGTCATCAACTGTAATTTGTG | | | 467 | ATCC25904Newman | |
|  | *sdrE* | GCAGCAGCGCATGACGGTAAAG | GTCGCCACCGCCAGTGTCATTA | | | 894 | Sanger476 | |
|  | *spa* | GCCAAAGCGCTAACCTTTTA | TCCAGCTAATAACGCTGCAC | | | 852 | NRS133 | |
|  | *hla* | CCAAGAATCTCTATCATATGGTC | GAAAGGTACCATTGCTGGTC | | | 391 | N315 | |
|  | *hlb* | AGTTGCAACACTTGCATTAGC | CTTCAGATTGTGTATGTGTACC | | | 202 | N315 | |
|  | *hld* | TGTTCACTGTGTCGATAATCC | CTCTCCTYACTGTYATTATACG | | | 342 | N315 | |
|  | *hlg* | ATGGATGTCACTCATGCC | GTATTTCCATTAAGTCCACCAG | | | 642 | N315 | |
|  | *psmα* | ATGGGTATCATCGCTGGCATCATTAAAGTTA | GTATTTCCATTAAGTCCACCAG | | | 406 | N315 | |
|  | *lukE* | GCAACTTTGTCAGTAGGACTG | GTCTACTTCACTGACATAACTC | | | 507 | COL | |
|  | *lukM* | TGGATGTTACCTATGCAACCTAC | GTTCGTTTCCATATAATGAATCACTAC | | | 795 | ATCC 31890 | |
|  | *pvl* | ATCATTAGGTAAAATGTCTGGACATGATCCA | GCATCAACTGTATTGGATAGCAAAAGC | | | 433 | JCSC2958 | |
| Primers for qRT-PCR | | | |  |  |  |  |  |
| *gyrB* | | CAAATGATCACAGCATTTGGTACAG | CGGCATCAGTCATAATGACGAT | | 91 | | | BA000018.3 |
| *hla* | | AATAACTGTAGCGAAGTCTGGTGAAA, | GCAGCAGATAACTTCCTTGATCCT; | | 81 | | | BA000018.3 |
| *psmα* | | TATCAAAAGCTTAATCGAACAATTC | CCCCTTCAAATAAGATGTTCATATC | | 176 | | | BA000018.3 |
| *pvl* | | CCAATAAATTCTGGATTGAAGTTACCT | GCTCAAGACAAAGCAACTTAAATGC | | 97 | | | AP009363.1 |
| *RNAⅢ* | | ATAGCACTGAGTCCAAGGAAACTAACT | GCCATCCCAACTTAATAACCATGT | | 83 | | | BA000018.3 |

**Table S2.** Clinical characteristics and molecular typing of CC59 isolates

|  |  |  | |  |  | **CA-MRSA** | | **HA-MRSA** | |
| --- | --- | --- | --- | --- | --- | --- | --- | --- | --- |
|  |  |  |  |  |  | **N(%,N/90)** | | **N(%,N/20)** | |
| ***Demographics*** | |  |  |  |  |  |  |  |  |
| Male |  | 67(60.9) |  |  |  | 55(61.1) |  | 12(60.0) |  |
| Age |  |  |  |  |  |  |  |  |  |
| Median age^*^ | | 0.4y |  |  |  | 0.3y |  | 1.1y |  |
| <1m |  | 23(20.9) |  |  |  | 21(23.2) |  | 2(10.0) |  |
| 1m-<1y |  | 45(40.9) |  |  |  | 38(42.2) |  | 7(35.0) |  |
| 1-<3y |  | 16(14.5) |  |  |  | 10(11.1) |  | 6(30.0) |  |
| 3-6y |  | 16(14.5) |  |  |  | 14(15.6) |  | 2(10.0) |  |
| ≥6y |  | 10(9.1) |  |  |  | 7(7.8) |  | 3(15.0) |  |
|  |  |  |  |  |  |  |  |  |  |
| ***Clinical Spectrum of Disease*** | | **No. of Cases** | **N (%) MRSA Type** |  |  |  |  |  |  |
| **Invasive Disease** | | **40(36.4)** |  |  |  | **29(32.2)** |  | **11(55.0)** |  |
| Severe pneumonia^+^ | | 23(20.9) |  |  |  | 18(20) |  | 5(25) |  |
|  |  |  | ST59-t437-IVa | |  | 15 | | 2 | |
|  |  |  | ST59-t441-IVa | |  | 1 | | 3 | |
|  |  |  | ST59-t437-V | |  | 1 | |  |  |
|  |  |  | ST338-t3590-V | |  | 1 | |  |  |
|  |  |  | ST59-t3523-IVa | |  |  |  |  |  |
| Bacteremia | | 7(6.4) |  |  |  | 7(7.8) |  | 0 |  |
|  |  |  | ST59-t437-IVa | |  | 4 | |  |  |
|  |  |  | ST59-t437-V | |  | 2 | |  |  |
|  |  |  | ST59-t3523-IVa | |  | 1 | |  |  |
| Cellulitis complicatedby bacteremia | | 2(1.8) |  |  |  | 2(2.2) |  | 0 |  |
|  |  |  | ST59-t3485-IVa | |  | 1 | |  |  |
|  |  |  | ST59-t5350-V | |  | 1 | |  |  |
| Suppurative peritonitis | | 1(0.9) |  |  |  | 1(1.1) |  | 0 |  |
|  |  |  | ST338-t437-V | |  | 1 | |  |  |
| Meningitis | | 3(2.7) |  |  |  | 1(1.1) |  | 2(10) |  |
|  |  |  | ST59-t437-IVa | |  | 1 | | 2 | |
| Osteomyelitis | | 3(2.7) |  |  |  |  |  | 3(15) |  |
|  |  |  | ST59-t437-IVa | |  |  |  | 3 | |
| Arthritis |  | 1(0.9) |  |  |  |  |  | 1(5.0) |  |
|  |  |  | ST59-t437-III | |  |  |  | 1 | |
| **Noninvasive disease** | | **70(63.6)** | |  |  | **61(67.7)** | | **90 (45)** |  |
| Pneumonia | | 29(26.4) |  |  |  | 24(26.7) |  | 5(25) |  |
|  |  |  | ST59-t437-IVa | |  | 19 | | 4 | |
|  |  |  | ST59-t437-V | |  | 3 | | 1 | |
|  |  |  | ST59-t441-IVa | |  | 1 | |  |  |
|  |  |  | ST375-t2270-IVa | |  | 1 | |  |  |
|  |  |  | ST59-t437-IVc | |  |  |  |  |  |
| Skin and soft tissue infections | | 32(29.1) |  |  |  | 29(32.2) |  | 3(15) |  |
|  | Cutaneous abscess | 13 |  |  |  | 13 |  |  |  |
|  |  |  | ST59-t437-IVa | |  | 10 | |  |  |
|  |  |  | ST59-t437-V | |  | 2 | |  |  |
|  |  |  | ST59-t441-IVa | |  | 1 | |  |  |
|  | Impetigo | 10 |  |  |  | 8 |  | 2 |  |
|  |  |  | ST59-t437-IVa | |  | 3 | | 1 | |
|  |  |  | ST59-t437-V | |  | 3 | | 1 | |
|  |  |  | ST338-t437-V | |  | 2 | |  |  |
|  |  |  | ST59-NT-III | |  |  |  |  |  |
|  |  |  | ST338-t437-III | |  |  |  |  |  |
|  | Neonatal omphalitis | 3 |  |  |  | 2 |  | 1 |  |
|  |  |  | ST59-t437-V | |  | 1 | | 1 | |
|  |  |  | ST59-t3523-IVa | |  | 1 | |  |  |
|  |  |  |  |  |  |  |  |  |  |
|  |  |  |  | |  |  | |  |  |
|  | Infected eczema | 2 |  |  |  | 2 |  | 0 |  |
|  |  |  | ST59-t437-IVa | |  | 2 | |  |  |
|  | Mastitis | 1 |  |  |  | 1 |  | 0 |  |
|  |  |  | ST59-t437-V | |  | 1 | |  |  |
|  | Traumatic wound | 3 |  |  |  | 3 |  | 0 |  |
|  |  |  | ST59-t437-IVa | |  | 1 | |  |  |
|  |  |  | ST338-437-V | |  | 2 | |  |  |
| Conjunctivitis |  | 2 (1.8) |  |  |  | 2 (2.2) |  | 0 |  |
|  |  |  | ST59-t437-V |  |  | 2 |  |  |  |
| Colonization |  | 7 (6.4) |  |  |  | 6(6.7) |  | 1(5.0) |  |
|  |  |  | ST59-t437-IVa |  |  | 5 |  | 1 |  |
|  |  |  | ST338-t3590-V |  |  | 1 |  |  |  |
|  |  |  |  |  |  |  |  |  |  |
| Total |  | 110 |  |  |  | 90 |  | 20 |  |
|  |  |  |  |  |  |  |  |  |  |

* m month, y year

+Severe pneumonia defined by a requirement for intensive care unit admission, necrotizing or cavitatoary infiltrates, or empyema
